# Concomitant Kawasaki Disease and Rotavirus Infection—More Than Just a Coincidence: A Case Report

**DOI:** 10.3390/tropicalmed8080388

**Published:** 2023-07-29

**Authors:** Mohammad Shukri Khoo, Adli Ali

**Affiliations:** 1 Department of Paediatrics, Faculty of Medicine, Universiti Kebangsaan Malaysia Children’s Specialist Hospital, The National University of Malaysia, Kuala Lumpur 56000, Malaysia; shukri.khoo@ukm.edu.my; 2 Research Centre, Universiti Kebangsaan Malaysia Children’s Specialist Hospital, The National University of Malaysia, Kuala Lumpur 56000, Malaysia; 3 Institute of IR4.0, The National University of Malaysia, Bangi 43600, Malaysia; 4 Infection and Immunology Health and Advanced Medicine Cluster, The National University of Malaysia, Kuala Lumpur 56000, Malaysia

**Keywords:** Kawasaki, rotavirus, complication, hyponatraemia, anasarca

## Abstract

The definitive role of viral infections, such as rotavirus, in causing Kawasaki disease (KD) remains uncertain. However, the intriguing observation of concomitant rotavirus infection and KD suggests a potential association. This study aimed to investigate this relationship. We reported a case of concomitant KD and rotavirus infection complicated by hyponatraemia and anasarca. For the systematic review, we used three large databases, namely PubMed, Ovid, and Scopus, to search articles with the terms “Kawasaki” and “rotavirus”. We also used Google Scholar as our secondary source. We included articles that fulfilled the following criteria: (i) articles reporting on children aged 18 and below; (ii) articles reporting on patients infected with rotavirus prior to or concomitant with KD; and (iii) articles written in English only. Three articles were included and analysed in combination with our reported patient. All patients exhibited gastrointestinal symptoms, including diarrhoea and vomiting, in addition to non-resolving fevers, which eventually manifested more signs and symptoms to support the diagnosis of KD. Stool samples from all patients revealed positive rotavirus antigens. Two patients (n = 2) were noted to have hyponatraemia and hypoalbuminaemia. Three (n = 3) manifested coronary artery abnormalities (CAA). Even though the relationship is not fully understood yet, it is known that the combination of these two pathologies can cause catastrophic immune responses and complications.

## 1. Introduction

Kawasaki disease (KD) is an acute febrile systemic vasculitis syndrome that affects children worldwide, especially in East Asia [1]. It causes significant morbidity and mortality due to its devastating complications, especially in the cardiovascular sequelae due to inflammatory injuries to the coronary arteries. To date, the exact cause of KD remains elusive, although infection is strongly suspected as a possible culprit [2]. However, the role of infection in KD is still being investigated by researchers around the globe. It is proposed to be the outcome of an atypical and exaggerated immune response that is triggered by infectious agents in genetically vulnerable individuals [2,3,4]. Rotavirus infection and KD have always sparked interest in their mysterious association. Interestingly, the development of hyponatraemia, anasarca, and coronary artery abnormalities (CAA) has been similarly mentioned in cases of concomitant rotavirus infection with KD. 

## 2. Materials and Methods

We reported a patient who was treated by our paediatric team at Hospital Canselor Tuanku Muhriz (HCTM), Kuala Lumpur, Malaysia. The patient’s medical records were retrieved, and information was gathered by accessing the patient’s physical and digital records.

**Figure 1:**
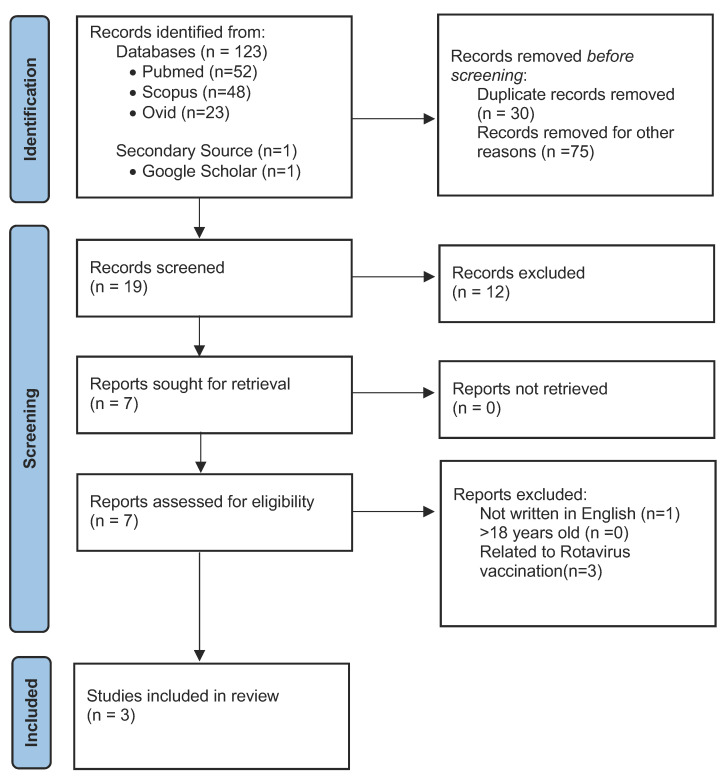
PRISMA flow diagram for our literature search.

Then we conducted a systematic review in compliance with the Preferred Reporting Items for Systematic Reviews and Meta-Analyses (PRISMA) guidelines. We utilised three large medical databases, namely PubMed, Ovid, and Scopus, to identify articles that include the terms “Kawasaki” and “rotavirus” from inception until October 2022. In addition, the Google Scholar search engine was used as a secondary source to look up possible missing relevant articles. We reviewed the articles based on the title and abstract. The screening process involved both researchers independently assessing the studies. The articles were then retrieved and reviewed thoroughly, and we included cases that fulfilled our inclusion criteria: (i) patients less than 18 years old; (ii) patients infected with rotavirus prior to or concurrent with KD; and (iii) articles written in English only. We excluded cases of KD following rotavirus vaccination and other viruses apart from rotavirus. The process of the literature search is summarised using the PRISMA flow diagram (Figure 1). The findings were then compiled and tabulated.

## 3. Case Report

A 4-month-old girl presented with diarrhoea and a persistent fever for 3 days, associated with poor oral intake. There was no other significant medical or surgical history. It was confirmed that the patient had not received the rotavirus vaccine. She was initially admitted for poor hydration due to acute gastroenteritis (AGE), and intravenous hydration and other methods of supportive management were initiated. Upon examination, macular rashes were observed over the limbs, trunk, and face. By day 6 of her illness, she began to demonstrate redness in both her eyes, cracked lips, and peeling of the periungual regions. She began to become more oedematous despite the persistent diarrhoea, and a physical examination revealed anasarca with the presence of multiple shotty cervical lymph nodes. Haematological investigations revealed mild anaemia (haemoglobin of 10.4 g/dL) and markedly increased total white cells of 47.2 × 10^9^/L (neutrophils predominant at 93% and lymphocytes at 6.7%). She was hyponatraemic (125 mmol/L), and liver function tests revealed the presence of hypoalbuminemia (18 g/L). Her C-reactive protein (CRP) was 20.78 mg/L, and her stool was positive for rotavirus antigen. An antistreptolysin O titre test was negative. As the fever persisted for more than 5 days, with the subsequent clinical signs documented, KD was diagnosed, and she was promptly treated with intravenous immune globulin (IVIG) at 2 g/kg over 12 h and oral acetylsalicylic acid at 80 mg/kg/day. No complications were observed during or after the treatment. An echocardiogram was performed, and a right coronary artery ectasia with a diameter of 4 mm was noted. Her general condition improved after the administration of IVIG. She was discharged after a week of admission. The patient remained well and asymptomatic with regular follow-ups for the coronary artery ectasia, which has remained unchanged since her discharge.

## 4. Results

Our search strategy yielded a total of 124 published articles. After removing 30 duplicates, we conducted title and abstract screenings, leading to the exclusion of 75 studies that were not suitable, were significantly unrelated to our research topic, and appeared to have been included in the search results by chance. This left us with 19 studies for full-text review. Upon careful evaluation, twelve studies were excluded because they were primarily focused on investigating other viruses or pathogens or exploring the relationship between KD and the rotavirus vaccine rather than focusing directly on the infection itself, resulting in a final review of seven published articles. Out of these, four articles were excluded due to non-compliance with the inclusion criteria. Finally, a total of three articles [5,6,7] were selected for inclusion in the review.

The review encompassed a total of four patients, including one newly reported case (our patient) and three previously reported cases. The age range of the patients varied from 3 months to 4 years, with two male (n = 2) and two female (n = 1) patients. None of the patients had any significant underlying diseases or syndromes prior to the onset of the presentation of acute KD.

All the patients presented acutely with gastrointestinal symptoms, including diarrhoea (n = 4) and vomiting (n = 3). Additionally, they exhibited persistent fevers (n = 4), accompanied by varying degrees of dehydration. Based on the initial symptoms, they were all diagnosed with AGE, and all patients were admitted for hydration and supportive management. During the later stages of the illness, mostly during the hospitalisation period, the classic signs and manifestations of KD developed. Among the reported cases, our patient was the only one who fulfilled the diagnostic criteria for KD, while the remaining cases were classified as incomplete or atypical KD. All cases were treated promptly with IVIG and acetylsalicylic acid. No patients received additional adjunctive therapies.

It was observed that all patients had a significantly increased white cell count with neutrophilia (n = 4). Thrombocytosis was present in three patients (n = 3), while information regarding platelet count was not reported in the case by D’Auria et al. [6]. All cases had high inflammatory markers, namely CRP and the erythrocyte sedimentation rate (ESR). Interestingly, both our patient and the case reported by D’Auria et al. [6] exhibited the atypical findings of hyponatraemia and hypoalbuminaemia as complications. The rotavirus antigen was detected and confirmed positive in all patients (n = 4). Echocardiography (ECHO) was carried out in all cases, revealing varying degrees of abnormalities in the coronary arteries in three patients (n = 3). These results are summarized in Table 1 and Table 2.

## 5. Discussion

KD is an acute febrile systemic vasculitis syndrome that predominantly affects children, with 85% of cases occurring in children under 5 years old [8]. The prevalence of KD is increasing worldwide, particularly in Asia. It is more prevalent in Japan, with an annual prevalence of 264.8 cases per 100,000 children younger than 5 years old [9]. Though the trend increases every year, to date, the main aetiology of KD has remained mystery since its first discovery in 1967. Various factors have been suggested as potential causes, including infectious agents such as viruses and bacteria, ethnicity, genetics, and exposure to cigarette smoke [1]. Evidence suggests that infection, particularly in genetically susceptible individuals, plays a significant role in the development of KD [10,11]. The impact of genetics is evident, as Asian children are affected by KD at a rate ten times higher than other populations [12].

Although extensive investigations have been conducted, not a single specific organism responsible for KD has been identified, even with advanced microorganism culture and serological techniques. The rotavirus vaccine RotaTeq^®^ was initially associated with an increased risk of KD during pre-marketing phase 3 clinical trials; however, the recent literature has shown no substantial association between the vaccine and KD [13,14,15]. Nevertheless, our particular interest for this study lay in investigating the potential association within the rotavirus infection itself as we have observed a few cases with similar characteristics, and there has always been a spark of interest in the significant association between these two pathologies. There are currently no specific prevalence data available regarding the occurrence of KD in relation to rotavirus infection in Malaysia or globally. It is possible that the occurrence of this combination is rare, which could explain the lack of prevalence reports on the topic. A study by Matsuno et al. [16] reported rotavirus capsomers were found in 74% of stool samples from patients with Kawasaki syndrome. Furthermore, from the serologic tests performed, a notable increase in the rotavirus antibody was found in 51% of the patients [16]. Given these findings, the co-occurrence of rotavirus infection and KD in the reported cases suggests more than just a coincidental relationship. We support the idea that this might be related to the outcome of rotavirus acting as a superantigen and eventually causing catastrophic systemic inflammation, as seen in KD.

Interestingly, a study by D’Auria et al. [6] also mentioned the development of hyponatraemia and anasarca in patients with KD following rotavirus infection. In both our reported case and the study by D’Auria et al. [6], it was suggested that the hyponatraemia and anasarca in these patients may be caused by more than just dehydration. Instead, they could be the results of severe inflammation triggered by the infection and the disease itself, leading to microvascular hyperpermeability [17,18,19]. This association is further supported by a case report of concomitant COVID-19 infection and KD by Jones et al. [20] in which the patient also experienced hyponatraemia and anasarca. Watanabe et al. [21] and Kil [22] proposed that the severity of inflammation is closely related to the occurrence of hyponatraemia in KD. This was further corroborated by a study by Nakamura et al. [23] that observed that a serum sodium concentration below 135 mmol/L might serve as an indicator for the potential development of giant coronary aneurysms associated with KD. The exact mechanism underlying these observations is still unknown, but Park et al. [18] suggested that IL-1 and IL-6 may play significant roles in the development of hyponatraemia in KD, particularly in association with the syndrome of inappropriate antidiuretic hormones (SIADH). This hypothesis was supported by Lim et al. [24], who observed higher levels of serum IL-1 and IL-6 in patients diagnosed with KD and hyponatraemia. The levels of antidiuretic hormone (ADH) were found to be proportional to the IL-1 and IL-6 levels, suggesting that they may be responsible for hyponatraemia in patients with KD. Additionally, a study showed elevated levels of IL-6 in children infected with rotavirus compared to healthy children [25]. Severe inflammation can promote microvascular hyperpermeability via vascular endothelial growth factor (VEGF) and hepatocyte growth factor (HGF), resulting in endothelial gap formation and subendothelial oedema, causing the extravasation of albumin [26,27], which may contribute to the development of anasarca. Moreover, due to increased cytokine levels in patients with KD, the production of protein in the liver can be disrupted, leading to severe hypoalbuminaemia and subsequent oedema [26,28].

Another intriguing observation is the occurrence of CAA in three of the patients included in this review. CAA is a well-recognised and serious complication of KD that is associated with significant morbidity and mortality, particularly if left untreated. Suzuki et al. have suggested that oedema may serve as an important risk factor for the development of coronary artery lesions, which can be attributed to the microvascular hyperpermeability discussed earlier. Furthermore, it is noted that a high CRP level increases the risk of CAA [29,30]. As CRP is a sensitive marker for inflammation, it can be deduced that a higher severity of inflammation in KD increases the risk of CAA.

Rotavirus can act like a superantigen and cause devastating complications, as observed in all the patients reviewed in this study. Furthermore, gastrointestinal symptoms like diarrhoea, vomiting, and abdominal pain are not uncommon in patients with atypical KD [31]. Therefore, it is important for clinicians to maintain a high level of clinical suspicion of KD in patients presenting with gastrointestinal symptoms and to not delay treatment if clinically indicated.

IVIG is the first-line treatment for KD [32,33]. However, clinicians are recommended to consider adjunctive treatment [34,35,36] like corticosteroids, infliximab, etanercept, plasmapheresis, and secondary IVIG infusion [1], especially in cases in which there is an increased risk of developing CAA. Further research is needed to clarify the role of adjuvant therapy in patients with concomitant KD and rotavirus infection.

Several limitations have been identified in this study. Firstly, the available literature on concomitant rotavirus infection and KD is limited in terms of the number of reported cases, and there are instances in which data are incomplete or missing from the reports. As a result, this study may not fully capture the comprehensive understanding of this disease. We came across a study that shares similarities with our cases; however, it is available only in Spanish. We acknowledge that our study might be susceptible to potential bias due to the exclusion of relevant studies conducted in languages other than English.

## 6. Conclusions

The association between KD and rotavirus infection goes beyond mere coincidence. While the exact mechanisms underlying this association remain unclear, it is evident that it can lead to significant complications such as hyponatraemia, anasarca, and, most importantly, CAA. Hence, its early and accurate diagnosis is crucial, followed by a timely initiation of treatment to prevent potential morbidity and mortality associated with this relationship.

## Figures and Tables

**Table 1:** Summary of patient demographics, initial presentations, KD diagnosis criteria, and management.

	Our Patient	Prashanth et al. [5]	D’Auria et al. [6]	Öksüz and İğde [7]
***Patient Demographics***				
Age	4 months	11 months	4 years	3 months
Gender	Female	Male	Male	Female
***Initial Presentation***				
Fever	+	+	+	+
Loose stool	+	+	+	+
Vomiting	−	+	+	+
***Criteria for KD***				
Fever > 5 days	+	+	+	+
Bilateral conjunctivitis	+	−	−	+
Oral changes	+	+	−	+
Extremity changes	+	−	−	−
Lymphadenopathy	+	+	+	−
Diffuse maculopapular rash	+	+	−	+
***Management***				
Treatment	IVIG 2 g/kg over 12 h and oral acetylsalicylic acid 80 mg/kg			

**Table 2:** Summary of patients’ laboratory investigations and echocardiography results.

	Our Patient	Prashanth et al. [5]	D’Auria et al. [6]	Öksüz and İğde [7]
*Blood investigations*				
Haemoglobin (g/dL)	10.4	9.2	N/A	10.7
White blood cells (10^9^/L)	47.2	12	14.5	21.7
Neutrophils (10^9^/L)	43.9 (93%)	(72%)	N/A	(69%)
Platelet (10^9^/L)	589	750	N/A	674
CRP (mg/L)	20.78	54	133	36
ESR (mm/h)	N/A	N/A	N/A	82
Sodium (mmol/L)	125	N/A	121	Normal
Potassium (mmol/L)	6.2	N/A	4.2	Normal
Urea (mmol/L)	2.2	N/A	13	Normal
Albumin (g/dL)	18	Normal	44.2	Normal
Creatinine (umol/L)	18	Normal	2.23	Normal
Bilirubin (umol/L)	6	Normal	N/A	Normal
ALP (IU/L)	99	Normal	N/A	Normal
ALT (IU/L)	17	Normal	N/A	Normal
*Microbial Investigation*				
Rotavirus antigen	Positive	Positive	Positive	Positive
*Other investigation*				
Echocardiography	Right coronary artery ectasia with a diameter of 4 mm.	NAD	Proximal part of the right coronary artery aneurysm with a diameter of 3.9 mm and a generalised dilation of the left coronary artery without pericardial effusion.	Dilatation of the left coronary artery with a diameter of 3.2 mm. Normal right coronary artery.

CRP: C-reactive protein; ESR: erythrocyte sedimentation rate; ALP: alkaline phosphatase; ALT: alanine aminotransferase; ASOT: anti-streptolysin O; NAD: no abnormalities detected; N/A: not available.

## Data Availability

The data presented in this study are available in the article.
